# Eradication Treatment of *Helicobacter pylori* Infection: Its Importance and Possible Relationship in Preventing the Development of Gastric Cancer

**DOI:** 10.5402/2012/935410

**Published:** 2012-06-13

**Authors:** Bruna Maria Roesler, Sandra Cecília Botelho Costa, José Murilo Robilotta Zeitune

**Affiliations:** ^1^Department of Clinical Medicine, Faculty of Medical Sciences, State University of Campinas (UNICAMP), 13.081-970 Campinas, SP, Brazil; ^2^Center of Diagnosis of Digestive Diseases, Faculty of Medical Sciences, State University of Campinas (UNICAMP), 13.081-970 Campinas, SP, Brazil

## Abstract

*Helicobacter pylori* is the most important carcinogen for gastric adenocarcinoma. Bacterial virulence factors are essential players in modulating the immune response involved in the initiation of carcinogenesis in the stomach; host genetic factors contribute to the regulation of the inflammatory response and to the aggravation of mucosal damage. In terms of environmental factors, salt intake and smoking contribute to the development of lesions. Various therapeutic schemes are proposed to eradicate *H. pylori* infection, which could potentially prevent gastric cancer, offering the greatest benefit if performed before premalignant changes of the gastric mucosa have occurred.

## 1. Introduction

The first isolation of *Helicobacter pylori* in the 1980s by Marshall and Warren [1] brought to the medical and scientific communities a new understanding of the pathogenesis of diseases that affect the digestive tract. Since then, *H. pylori* infection has been associated with the development of acute and chronic gastritis, atrophic gastritis, peptic ulcer disease, gastric mucosa-associated lymphoid tissue (MALT) lymphoma, and gastric adenocarcinoma [2–4]. 

All patients with *H. pylori* infection have histological gastritis, which corresponds to classical chronic gastritis and is characterized by the infiltration of neutrophils and other inflammatory cells. However, most patients are asymptomatic for life, while only some will come to develop a digestive disease [5]. 

Furthermore, when the relationship between *H. pylori* infection and chronic gastritis was established, investigators began to take interest in the causal role of the bacterium in gastric cancer [6]. On the basis of numerous subsequent epidemiological studies, *H. pylori* infection was shown to be associated with an increased risk of gastric adenocarcinoma development [5, 7]. Evidence that the presence of *H. pylori* increases the risk of developing gastric cancer through atrophy and intestinal metaplasia has also been reported [8, 9], suggesting that *H. pylori*-positive patients develop these conditions in greater proportion than control subjects. Consequently, in 1994, the World Health Organization's International Agency for Research on Cancer concluded that *H. pylori* has a causal link with gastric carcinogenesis and was defined as a type I carcinogen, a definite human carcinogen [10]. 

It is known that gastric cancer involves the interaction of three major factors: the agent (in the great part of the cases, *H. pylori*) and its pathogenicity, the characteristics of the host, and the external environment [10–14].

Specifically regarding *H. pylori* infection, there are some studies indicating that the eradication of the microorganisms in the system could reduce the incidence of gastric cancer in patients without precancerous lesions or, when lesions are present, that the eradication may or may not reduce this incidence [15]. Also, when the eradication is done after endoscopic mucosal resection in patients with early gastric adenocarcinoma, it could decrease the recurrence of metachronous gastric cancer in some patients [16]. 

Based on these observations, in this paper, we will attempt to provide a comprehensive overview of the principal concepts in the management of *H. pylori* infection, the lines of treatment and the importance of eradication of this bacterium in precancerous lesions and in early gastric cancer. 

## 2. Management Guidelines

Various guidelines for the management of *H. pylori* infection worldwide are available. Among these, the latest ones are the “Maastricht III Consensus Report” (2005) [17], the “American College of Gastroenterology Guideline on the Management of *Helicobacter pylori* Infection” (2007) [18], and the “Second Asia-Pacific Consensus Guidelines for *Helicobacter pylori* Infection” (2009) [19].

Generally, the eradication of *H. pylori* in adults is recommended when the bacterium is present in the gastric mucosa. However, a discussion may arise about whether or not to recommend specific treatment in asymptomatic individuals that receive positive diagnoses for *H. pylori* in routine exams. In these cases, patients should be advised about the therapy, the adverse effects resulting from the use of the chosen medications, and the importance of *H. pylori* eradication in order to prevent some gastric diseases, such as peptic ulcer disease and gastric cancer. The same approach should be taken with respect to patients with functional dyspepsia. *H. pylori* eradication offers modest but significant benefits in these patients [20], and economic modeling suggests that this benefit is cost effective [21]. 

Nowadays, eradication of *H. pylori* is recommended for patients with gastroduodenal ulcers, MALT lymphoma, in individuals with chronic use of nonsteroidal anti-inflammatory drugs (NSAIDs), and proton pump inhibitors (PPIs), in patients with atrophic gastritis and erosive duodenitis and in cases of gastric adenocarcinoma subjected to surgical treatment. First-degree relatives of patients with early or advanced distal gastric adenocarcinoma have to be evaluated for *H. pylori*, and those who test positive have to be submitted to eradication treatment. 

As for gastroesophageal reflux disease, eradication of *H. pylori* does not cause it and does not exacerbate symptoms in patients with it either when untreated or in those receiving PPI maintenance treatment [17, 22]. International consensus statements diverge, with European [17, 23] and Canadian [24] guidelines recommending treatment and US guidelines not recommending treatment. In the Asia-Pacific region, where reflux disease is less common while ulcer disease and gastric cancer are more common, it is recognized that the likelihood of and benefit in treating *H. pylori* infection will be commensurately greater [19].

 Furthermore, among the extradigestive diseases that have been associated with *H. pylori* infection, the eradication of the bacterium is recommended in patients with unexplained iron deficiency anemia and in patients with chronic idiopathic thrombocytopenic purpura, when all other known causes have been carefully excluded [17, 23].

## 3. Treatment

After a long evaluation period for multiple therapeutic regimens for the eradication of *H. pylori* in infected individuals, testing single medication regimens as well as the association of two, three or four medications, some conclusions were reached, among them that treatments should use at least three associated drugs.

Knowledge about the structural characteristics and the pharmacokinetics of each chosen drug is also important. *H. pylori* has a thick glycocalyx that partially inhibits the drug's action, which is also hampered by the bacterium's adherence to the gastric epithelium. Furthermore, it is important that each medication that acts directly on the bacterium be able to dissolve rapidly in the stomach and remain stable across a wide pH range, especially in an acidic environment. It is also necessary that the active substance has appropriate dimensions and ionic charge in order to allow it to enter into the mucous layer and come close to the gastric mucosa. Moreover, the medications with systemic action have to be absorbed by the stomach and small intestine in order to be secreted by the gastric mucosa. 

Included in the therapeutic regimen, gastric acid secretion inhibitors contribute to increase *H. pylori* eradication rates. They can also relieve the adverse effects of other drugs and, consequently, help to eliminate pain symptoms more quickly. Furthermore, these drugs probably facilitate the bactericidal action of antimicrobials by increasing intragastric pH. In turn, the proton pump inhibitors (e.g. omeprazole, lansoprazole, and pantoprazole) have a bacteriostatic effect and cause the increase of the half-life of some antimicrobial drugs in plasma. 

Therapeutic options usually include clarithromycin, metronidazole (or tinidazole), amoxicillin, furazolidone, tetracycline (tetracycline phosphate complex, oxytetracycline, and doxycycline), colloidal bismuth subcitrate, ranitidine bismuth citrate (H_2_ receptor antagonist), and a PPI (omeprazole, lanzoprazole, or pantoprazole), along with other antimicrobial agents. 

Table 1 shows the main recommended regimens for *H. pylori* eradication, including the most used regimens with clarithromycin and amoxicilin with a PPI, and the most recent therapies, as levofloxacin-based triple therapy, the levofloxacin-based quadruple therapy, and the rifabutin-based triple therapy. Usually, the duration of treatment is seven to ten days but may reach up to 14 days, as appropriate. 

According to the “Maastricht III Consensus Report” [17], both three- and four-drug regimens are considered first-line treatments. Standard triple therapy consisting of a PPI, clarithromycin, and amoxicillin or metronidazole is more successful if extended to more than seven days. Furthermore, increased resistance to antibiotics used in the PPI triple therapy, a phenomenon that has been observed worldwide, needs to be considered in the selection of treatment. For instance, the increase of clarithromycin resistance has been found to be one of the main risk factors for treatment failure in most countries [25, 26]. 

Recently, sequential treatment consisting of five days of a PPI plus amoxicillin, followed by five additional days of a PPI plus clarithromycin and tinidazole, has been shown to be better than the combination of a PPI plus amoxicillin and clarithromycin for seven days [27, 28]. Novel first-line anti-*H. pylori* therapies in 2011 include sequential therapy, concomitant quadruple therapy, hybrid (dual-concomitant) therapy, and bismuth-containing quadruple therapy.

Moreover, in cases of patients for whom there was a failure of the standard triple therapy, a bismuth-containing quadruple therapy comprising a PPI, bismuth, tetracycline, and metronidazole can be employed as rescue treatment. Triple therapy combining a PPI, levofloxacin, and amoxicillin has been proposed as an alternative to the standard rescue therapy. This salvage regimen can achieve a higher eradication rate than bismuth-containing quadruple therapy in some regions and has fewer adverse effects. 

Levofloxacin-based triple therapy consisting of levofloxacin (500 mg, once daily), amoxicillin (1g, twice daily), and a PPI (standard dose, twice daily) represents an encouraging strategy for second-line therapy. The second-line therapy for patients who fail to eradicate *H. pylori* with the new first-line therapies (such as sequential therapy, concomitant therapy, or hybrid therapy) remains unclear [29]. Meanwhile, a recent study showed that a levofloxacin-based triple therapy with lanzoprazole (30 mg, twice daily), levofloxacin (250 mg, twice daily), and amoxicillin (1g, twice daily) achieved a high eradication rate in patients who had failed to clear *H. pylori* with sequential therapy [29, 30]. 

With respect to the third-line therapy, the Maastricht III Consensus Report recommends the use of bacterial cultures with antimicrobial sensitivity tests to select antibiotics for third-line regimens [17]. The antituberculous agent Rifabutin (150 mg, twice a day) is an option that can be administered with a PPI (standard dose, twice a day) and amoxicillin (1 g, twice a day) for ten to 14 days to eradicate *H. pylori* [31]. However, it has to be considered that serious adverse events can occur with rifabutin therapy, in addition to the development of more resistant strains of *Mycobacterium tuberculosis* and *Mycobacterium avium* [29]. 

Another possible treatment is the use of furazolidone, with a PPI and clarithromycin or tetracycline, regimens that have been utilized in Brazil. However, due to the possible genotoxic and carcinogenetic effects, furazolidone use is not approved in developed countries [32, 33].

Finally, considering the various and numerous therapeutic regimens and the differences in the eradication rates in different countries and even in regions within a country, it is essential to use medications that have been tested and that have been proven effective for the target population. 

## 4. *H. pylori* Eradication and Prevention of Gastric Cancer

### 4.1. Gastric Cancer and Its Classification

Gastric cancer remains a major global health problem [34] and, despite the decreasing incidence and mortality rates observed worldwide over the last 50 years, it still ranks as a leading cause of cancer-related deaths in many parts of the world [35]. Because the symptoms are often absent or nonspecific in the early stages of the disease, diagnosis of gastric cancer is usually made in advanced stages, when curative options are limited [36].

The vast majority of gastric cancers are adenocarcinomas, which can be generally divided into two types: the intestinal and the diffuse [37], which correspond, respectively, to the well-differentiated type and to the poorly-differentiated type in the Japanese classification [5].

The diffuse type is often associated with familial distribution and hereditary genetic alterations [38] and is developed in the stomach following chronic inflammation, especially in the cardia [35]. The causative germ-line mutation has been identified to be CDH-1, the e-cadherin gene, and this mutation represents an initial step in the process of downstream gene activation leading towards further increases in proliferation and cancer formation [39]. 

The intestinal type is thought to be preceded by a sequence of precursor lesions [40], the basic components of which are chronic inflammation of the gastric mucosa, which slowly progresses through the premalignant stages of atrophic gastritis, intestinal metaplasia, and dysplasia to gastric cancer [41]. While most studies claim that *H. pylori* infection is only related to distal gastric cancer, a high prevalence in patients with proximally located adenocarcinomas has recently been demonstrated [39]. Approximately 80% of patients with proximal gastric cancer reveal positive evidence for actual or past infection with *H. pylori* if correct allocation of the primary tumor is performed, and adenocarcinomas of the distal esophagus are strictly excluded [42]. 

Finally, the risk for gastric carcinogenesis by *H. pylori* infection is equal in the intestinal and diffuse types of gastric cancer [42, 43]. 

### 4.2. Early and Advanced Gastric Adenocarcinomas

Patients diagnosed in an early stage of the cancer present an excellent prognosis, with a five-year survival rate greater than 90%. In cases with advanced lesions, gastric cancer carries a poor prognosis, with an overall five-year survival rate of less than 20% [44]. 

Early gastric cancer is defined as an adenocarcinoma that is confined to the mucosa or submucosa, irrespective of lymph-node invasion. Part of early gastric cancers is believed to go through a life cycle consisting of ulcerations [45]. Nevertheless, some early lesions rapidly progress to advanced stages, and these cases comprise one of the principal questions concerning gastric carcinogenesis. 

Advanced gastric cancer, in turn, is defined as one that infiltrates the muscle layer along with all the other various layers of tissue [46]. Concerning the differences between the early and advanced stages of the disease, research has suggested that there is a biological continuum from benign disease to early, followed by advanced cancer. The point at which this sequence becomes irreversible has yet to be established [45]. Additionally, some questions about the development of the cancer remain unanswered: why would any tumor remain at an early stage for years without treatment while others become advanced in a short time? Have *H. pylori* virulence factors influenced this alteration? What about the host characteristics and environmental factors?

As written before, gastric carcinogenesis involves the interaction between an etiologic agent (for the most part, *H. pylori*), host characteristics, and the external environment. 

#### 4.2.1. *H. pylori* Virulence Factors

The strain-specific genes found in the comparison of sequenced strains are consistent with earlier studies that demonstrated the high diversity of the *H. pylori* genome [47–49]. Consequently, this high level of genetic diversity can be an important factor in its adaptation to the host stomach and for the clinical outcome of the infection although this aspect remains unclear. 

Many virulence genes of *H. pylori* have been reported to determine clinical outcomes; among those of potential significance, especially with regards to gastric cancer, are the cytotoxin-associated gene A (cagA), the cytotoxin-associated gene T (cagT), the vacuolating cytotoxin gene (vacA), and the outer inflammatory protein gene (oipA).

Both the cagA and cagT genes are located in the cag pathogenicity island (cagPAI), a genetic locus that encodes a type IV secretion system [50, 51]. Infection with cagA-positive strains has been associated with higher degrees of inflammation of the gastric mucosa; consequently, the gene seems to play an important role in the development of gastric cancer, being crucial to the formation of the precancerous lesions present in cases of intestinal type gastric adenocarcinoma [7, 9, 52, 53]. Upon delivery into host cells, CagA protein leads to dephosphorylation of host cell proteins and morphological changes in the cell [54, 55]. Additionally, CagA has been shown to interfere with *β*-catenin signaling [56, 57] and apical-junctional complexes [58], events that have been linked to increased cell motility and oncogenic transformation in a variety of models [51, 59, 60]. The cagT gene, in turn, is a homologue of *A*. *tumefaciens *virB7, and its role in the pathogenesis of gastrointestinal diseases has not been completely ascertained. Nonetheless, this gene has been considered an essential component in the IV secretion system and has been associated with peptic ulcer disease [61] and gastric cancer [62]. 

With respect to the VacA virulence factor, in addiction to inducing vacuolation, it can induce multiple cellular activities, including membrane-channel formation and cytochrome *c *release from mitochondria leading to apoptosis, and can bind to cell-membrane receptors, followed by the initiation of a proinflammatory response; it can also inhibit T-cell activation and proliferation [63–66]. The vacA gene presents three regions—s (signal), I (intermediate), and m (middle)—that have been associated with its cytotoxic activity [67–69]. For instance, studies have reported that individuals infected with s1 or m1 *H. pylori* strains have an increased risk of peptic ulcer or gastric cancer compared with individuals infected with s2 or m2 strains [70]. With respect to the intermediate region, classified in i1 and i2, it was shown that the i1 type is more pathogenic than i2 [69]. 

Finally, the OipA is an important virulence factor associated with enhanced interleukin-8 secretion and increased inflammation *in vitro* [71]. This gene is located in a chromosomal region close to the cagPAI and is present both as functional and nonfunctional. OipA functions as an adhesive and is reported to be involved in the attachment of *H. pylori* to gastric epithelial cells *in vitro* [72, 73], playing an important role in bacterial colonization. Its presence has been associated with the increase of Interleukin-8 in gastric cancer cells [74]. 

#### 4.2.2. Host Susceptibility

Polymorphisms in a wide variety of genes that are present within a significant proportion of the normal population may modify the effect of environmental exposure. These gene-environmental interactions could explain the high variation in the incidence of gastric cancer observed around the world [75]. Among these genes, for example, there are cytokine genes (TNF-*α*, IL-10, IL-8, and IFN-*γ*) involved in the adaptive immune system [76, 77] and pattern recognition factors (TLR-4, NOD-1, and NOD-2) involved in initiating the innate immune system [78, 79]. 

Host-related factors for the development of disease can indicate genetic susceptibility (or resistance) or acquired influences, which may stimulate defenses of the host against environmental carcinogens like *H. pylori* [80]. The relationship between host genetic polymorphisms (for instance, in the IL-1) and bacterial virulence factors appears to have a crucial role in the development of the cancer, especially in infections with cagA-positive, vacA s1m1, and oipA-positive strains.

#### 4.2.3. Environmental Factors

Environmental factors, particularly diet and smoking, play an important role in the pathogenesis of gastric cancer. Diet rich in complex carbohydrates, salt, pickled or smoked foods, dried fish, and cooking oil has been linked with an increased risk, while diet rich in fresh fruits and vegetables has been associated with a low risk of gastric cancer [75, 81].

Smoking also represents an important factor in gastric cancer development. A large study that included smoking men demonstrated an increased risk for the development of differentiated-type distal gastric cancer [82]. In Japan, a study offered persuasive evidence that tobacco smoking moderately increases the risk of gastric cancer among the Japanese population [83]. Another study that analyzed forty-two articles and compared current smokers and nonsmokers provided solid evidence to classify smoking as the most important behavioral risk factor for gastric cancer [84]. Finally, a recent study also concluded that smoking is an important factor associated with the risk of developing gastric cancer [85].

## 5. Eradication of *H. pylori* Infection and Gastric Cancer

### 5.1. Can Reversion of Precancerous Lesions Be Achieved by *H. pylori* Eradication?

The risk of gastric cancer is related to the severity and extent of atrophy, intestinal metaplasia, and dysplasia; the main question concerning the development of the cancer is whether eradication of *H. pylori* can reverse these precancerous lesions and interrupt the process of gastric carcinogenesis. 

Two randomized studies, the first with a five-year followup and the second with a one-year followup, observed that *H. pylori *eradication was beneficial in preventing progression of atrophy and intestinal metaplasia of the gastric mucosa [86, 87]. Similarly, one study carried out in a high-risk population suggested that effective anti-*H. pylori* treatment and dietary supplementation with antioxidant micronutrients may interfere with the precancerous process, mostly by increasing the rate of regression of cancer precursor lesions, and may be an effective strategy to prevent gastric carcinoma [88]. 

Still, a long-term follow-up study found that preneoplastic gastric lesions regress at a rate equal to the square of the time in patients rendered free of *H. pylori* infection, which also suggests that patients with preneoplastic gastric lesions should be treated and cured of their *H. pylori* infection [89]. These results are supported by others, both retrospective and prospective, that suggest that the eradication of *H. pylori* is important to prevent the development of gastric cancer [90–93]. 

However, despite a large number of studies demonstrating that *H. pylori* eradication has the potential to prevent gastric cancer development, there is a limitation to this claim: once preneoplastic changes have occurred, the prevention of further progression of invasive cancer through *H. pylori* eradication is less likely. One study carried out in 2004 was the first to indicate that bacterium eradication is not a guarantee for preventing gastric cancer in patients with chronic gastritis and existing preneoplastic changes [94]. 

Another critical aspect of the benefits of *H. pylori* eradication in the prevention of gastric cancer development is the timing for its occurrence. For instance, in a study carried out in Japanese patients with peptic ulcer disease, the incidence of gastric cancer was found to be 1.24% for those receiving *H. pylori* eradication therapy and 2.56% for those not, in a 5.6-year followup [95].

Consequently, it is obvious that there is a point of no return, after which curing the *H. pylori* infection no longer offers an effective prevention of gastric cancer [94, 96–98]. 

### 5.2. Eradication of *H. pylori* and Early Gastric Cancer: Is It Important and Possible to Prevent the Cancer Recurrence? 

For cancers detected early, endoscopic mucosal resection can conserve the noncancerous gastric mucosa, but it cannot eliminate the problem of recurrence of metachronous gastric cancer [99]. A study published in 2008 [100] suggested that prophylactic eradication of *H. pylori* in a high-risk population can substantially reduce gastric cancer rates. In this study, a randomized control trial following endoscopic resection of early gastric cancer, a group of patients was subjected to *H. pylori* eradication treatment and monitored at different time intervals. At three years, metachronous gastric cancer had developed in 9 of the 255 patients in the eradication group, while, in the control group, these lesions occurred in 24 of 250 patients. 

However, not all treated patients benefited, although the experimental data for humans are thin, they do support the notion that *H. pylori* causes cancer and that its treatment provides some benefit [98]. 

A very recent study carried out in patients with early-stage gastric cancer demonstrates that *H. pylori* eradication does not reduce the incidence of metachronous gastric cancer, bearing out that the eradication needs to be performed before the progression of the atrophy of the gastric mucosa [101]. Also emphasizing this aspect, another study concluded that gastric cancer development after eradication may have a carcinogenic pathway similar to that of cancer with *H. pylori* infection though macroscopic/biological features may be modified by the eradication therapy [102].

Results obtained from studies done with animal models suggest that eradication at an early stage might be effective in preventing carcinogenesis. Unfortunately, few of the animal models had results comparable to those of humans, as they were not infected for periods similar to those necessary for the development of cancer in humans [103].

## 6. Conclusions

Understanding of gastric carcinogenesis has advanced considerably over the past decades, especially with regards to insights into the role of *H*. *pylori* infection and the progression of chronic gastritis from premalignant stages to gastric cancer. Based on the review presented here, although controversy still remains as to whether eradication halts progression or can even cause regression of premalignant gastric lesions, it can be concluded that eradication of *H. pylori* infection has the potential to reduce the risk of gastric cancer development. Furthermore, the optimal time to eradicate the bacterium is before the development of preneoplastic lesions such as atrophic gastritis and intestinal metaplasia. Along with new therapeutic combinations, there is also a need to identify subjects most at risk for cancer from their genetic susceptibility and their infection with *H. pylori* genotypes of greater carcinogenic potential. Finally, early diagnosis is essential to ensure the best outcome of treatment, preventing the development and the worsening of the gastric cancer. 

## Figures and Tables

**Table 1:** Recommended regimens for the treatment of *H. pylori* infection in adults.

Treatment duration (days)	Recommended regimens (all drugs administered orally)
7–10	Clarithromycin (500 mg, b.i.d.) + amoxicillin (1 g, b.i.d.) + ranitidine bismuth citrate (400 mg, b.i.d.)
7–10	Clarithromycin (500 mg, b.i.d.) + amoxicillin (1 g, b.i.d.) + ranitidine bismuth citrate (400 mg, b.i.d.) + omeprazole (20 mg, b.i.d.) [or lanzoprazole (30 mg, b.i.d.) or pantoprazole (40 mg, b.i.d.)]
14	Metronidazole (400 to 500 mg, t.i.d. or q.i.d.) + colloidal bismuth subcitrate (120 mg, q.i.d.) + tetracycline (basic phosphate or oxytetracycline) (500 mg, q.i.d.)
7–10	Metronidazole (400 to 500 mg, b.i.d. or t.i.d.) + amoxicillin (500 mg, b.i.d. or t.i.d.) + omeprazole (20 mg, b.i.d.) [or lanzoprazole (30 mg, b.i.d.) or pantoprazole (40 mg, b.i.d.)]
4–7	Metronidazole (400 to 500 mg, t.i.d. or q.i.d.) + colloidal bismuth subcitrate (120 mg, q.i.d.) + tetracycline (basic phosphate or oxytetracycline) (500 mg, q.i.d.) + omeprazole (20 mg, b.i.d.) [or lanzoprazole (30 mg, b.i.d.) or pantoprazole (40 mg, b.i.d.)]
7	Clarithromycin (500 mg, b.i.d.) + metronidazole (400 to 500 mg, b.i.d.) + ranitidine bismuth citrate (400 mg, b.i.d.)
7	Clarithromycin (500 mg, b.i.d.) + metronidazole (400 to 500 mg, b.i.d.) + omeprazole (20 mg, b.i.d.) [or lanzoprazole (30 mg, b.i.d.) or pantoprazole (40 mg, b.i.d.)]
7	PPI (standard dose, q.d.) + clarithromycin (500 mg, b.i.d.) + furazolidone (200 mg, b.i.d.)^∗^
7	PPI (standard dose, q.d.) + furazolidone (200 mg, t.i.d.) + tetracycline (500 mg, q.i.d.)^∗^
7	Bismuth (standard dose, b.i.d.) + tetracycline (500 mg, b.i.d.) + furazolidone (200 mg, b.i.d.) + PPI (standard dose, b.i.d.) or ranitidine (standard dose, b.i.d.)^∗^
10	Levofloxacin (500 mg, q.d.) + amoxicillin (1 g, b.i.d.) + PPI (standard dose, b.i.d.)
10	Levofloxacin (500 mg, q.d.) + amoxicillin (500 mg, q.i.d.) + PPI (standard dose, b.i.d.) + bismuth (standard dose, q.i.d.)
14	Rifabutin (150 mg, b.i.d.) + amoxicillin (500 mg, b.i.d.) + PPI (standard dose, b.i.d.)

^∗^Treatments used in Brazilian patients.

## References

[1] Marshall BJ, Warren JR (1984). Unidentified curved bacilli in the stomach of patients with gastritis and peptic ulceration. *Lancet*.

[2] Yamada T, Searle JG, Ahnen D (1994). *Helicobacter pylori* in peptic ulcer disease. *The Journal of the American Medical Association*.

[3] Uemura N, Okamoto S, Yamamoto S (2001). *Helicobacter pylori* infection and the development of gastric cancer. *New England Journal of Medicine*.

[4] Wotherspoon AC, Ortiz-Hidalgo C, Falzon MR, Isaacson PG (1991). *Helicobacter pylori*-associated gastritis and primary B-cell gastric lymphoma. *Lancet*.

[5] Sugiyama T, Asaka M (2004). *Helicobacter pylori* infection and gastric cancer. *Medical Electron Microscopy*.

[6] Herrera V, Parsonnet J (2009). *Helicobacter pylori* and gastric adenocarcinoma. *Clinical Microbiology and Infection*.

[7] Parsonnet J, Friedman GD, Orentreich N, Vogelman H (1997). Risk for gastric cancer in people with CagA positive or CagA negative *Helicobacter pylori* infection. *Gut*.

[8] Asaka M, Kimura T, Kato M (1994). Possible role of *Helicobacter pylori* infection in early gastric cancer development. *Cancer*.

[9] Kuipers EJ, Perez-Perez GI, Meuwissen SGM, Blaser MJ (1995). *Helicobacter pylori* and atrophic gastritis: importance of the cagA status. *Journal of the National Cancer Institute*.

[10] International Agency for Research on Cancer (1994). *Schistosomes, Liver Flukes and Helicobacter pylori*.

[11] Kabir S, Grant C, Daar AS (1995). Serum levels of interleukin-1, interleukin-6 and tumour necrosis factor-alpha in patients with gastric carcinoma. *Cancer Letters*.

[12] El-Omar EM, Carrington M, Chow WH (2000). Interleukin-1 polymorphisms associated with increased risk of gastric cancer. *Nature*.

[13] Correa P, Piazuelo MB, Camargo MC (2004). The future of gastric cancer prevention. *Gastric Cancer*.

[14] Ando T, Goto Y, Ishiguro K (2007). The interaction of host genetic factors and *Helicobacter pylori* infection. *Inflammopharmacology*.

[15] Kabir S (2009). Effect of *Helicobacter pylori* eradication on incidence of gastric cancer in human and animal models: underlying biochemical and molecular events. *Helicobacter*.

[16] Uemura N, Mukai T, Okamoto S (1997). Effect of *Helicobacter pylori* eradication on subsequent development of cancer after endoscopic resection of early gastric cancer. *Cancer Epidemiology Biomarkers and Prevention*.

[17] Malfertheiner P, Megraud F, O’Morain C (2007). Current concepts in the management of *Helicobacter pylori* infection: the Maastricht III Consensus Report. *Gut*.

[18] Chey WD, Wong BCY (2007). American College of Gastroenterology guideline on the management of *Helicobacter pylori* infection. *American Journal of Gastroenterology*.

[19] Fock KM, Katelaris P, Sugano K (2009). Second Asia-Pacific Consensus Guidelines for *Helicobacter pylori* infection. *Journal of Gastroenterology and Hepatology*.

[20] Moayyedi P, Deeks J, Talley NJ, Delaney B, Forman D (2003). An update of the cochrane systematic review of *Helicobacter pylori* eradication therapy in nonulcer dyspepsia: resolving the discrepancy between systematic reviews. *American Journal of Gastroenterology*.

[21] Moayyedi P, Soo S, Deeks J (2000). Systematic review and economic evaluation of *Helicobacter pylori* eradication treatment for non-ulcer dyspepsia. *BMJ*.

[22] Laine L, Sugg J (2002). Effect of *Helicobacter pylori* eradication on development of erosive esophagitis and gastroesophageal reflux disease symptoms: a post hoc analysis of eight double blind prospective studies. *American Journal of Gastroenterology*.

[23] Caselli M, Zullo A, Maconi G (2007). "Cervia II Working Group Report 2006": guidelines on diagnosis and treatment of *Helicobacter pylori* infection in Italy. *Digestive and Liver Disease*.

[24] Veldhuyzen van Zanten SJO, Bradette M, Chiba N (2005). Evidence-based recommendations for short- and long-term management of uninvestigated dyspepsia in primary care: an update of the Canadian Dyspepsia Working Group (CanDys) clinical management tool. *Canadian Journal of Gastroenterology*.

[25] Mégraud F, Lamouliatte H (2003). Review article: the treatment of refractory *Helicobacter pylori* infection. *Alimentary Pharmacology and Therapeutics*.

[26] McMahon BJ, Hennessy TW, Bensler JM (2003). The relationship among previous antimicrobial use, antimicrobial resistance, and treatment outcomes for *Helicobacter pylori* infections. *Annals of Internal Medicine*.

[27] Zullo A, Vaira D, Vakil N (2003). High eradication rates of *Helicobacter pylori* with a new sequential treatment. *Alimentary Pharmacology and Therapeutics*.

[28] De Francesco V, Zullo A, Margiotta M (2004). Sequential treatment for *Helicobacter pylori* does not share the risk factors of triple therapy failure. *Alimentary Pharmacology and Therapeutics*.

[29] Chuah S-K, Tsay F-W, Hsu P-I (2011). A new look at anti-*Helicobacter pylori* therapy. *World Journal of Gastroenterology*.

[30] Pontone S, Standoli M, Angelini R, Pontone P (2010). Efficacy of *H. pylori* eradication with a sequential regimen followed by rescue therapy in clinical practice. *Digestive and Liver Disease*.

[31] Gisbert JP, Calvet X, Bujanda L, Marcos S, Gisbert JL, Pajares JM (2003). ’Rescue’ therapy with rifabutin after multiple *Helicobacter pylori* treatment failures. *Helicobacter*.

[32] De Francesco V, Ierardi E, Hassan C, Zullo A (2009). Is furazolidone therapy for *Helicobacter pylori* effective and safe?. *Digestive Diseases and Sciences*.

[33] Zullo A, Ierardi E, Hassan C (2012). Furazolidone-based therapies for *Helicobacter pylori* infection: a pooled-data analysis. *Saudi Journal of Gastroenterology*.

[34] Malfertheiner P, Bornschein J, Selgrad M (2010). Role of *Helicobacter pylori* infection in gastric cancer pathogenesis: a chance for prevention. *Journal of Digestive Diseases*.

[35] Nardone G, Rocco A, Malfertheiner P (2004). Review article: *Helicobacter pylori* and molecular events in precancerous gastric lesions. *Alimentary Pharmacology and Therapeutics*.

[36] Hohenberger P, Gretschel S (2003). Gastric cancer. *Lancet*.

[37] Lauren P (1965). The two histological main types of gastric carcinoma: diffuse and so-called intestinal-type carcinoma. *Acta Pathologica et Microbiologica Scandinavica*.

[38] Barber M, Fitzgerald RC, Caldas C (2006). Familial gastric cancer—aetiology and pathogenesis. *Best Practice and Research: Clinical Gastroenterology*.

[39] Bornschein J, Malfertheiner P (2011). Gastric carcinogenesis. *Langenbeck’s Archives of Surgery*.

[40] Correa P, Haenszel W, Cuello C (1975). A model for gastric cancer epidemiology. *Lancet*.

[41] Correa P, Houghton J (2007). Carcinogenesis of *Helicobacter pylori*. *Gastroenterology*.

[42] Bornschein J, Selgrad M, Warnecke M, Kuester D, Wex T, Malfertheiner P (2010). *H. pylori* infection is a key risk factor for proximal gastric cancer. *Digestive Diseases and Sciences*.

[43] Hansen S, Vollset SE, Derakhshan MH (2007). Two distinct aetiologies of cardia cancer; evidence from premorbid serological markers of gastric atrophy and *Helicobacter pylori* status. *Gut*.

[44] Bowles MJ, Benjamin IS (2001). ABC of the upper gasrointestinal tract: cancer of the stomach and pancreas. *BMJ*.

[45] Everett SM, Axon ATR (1998). Early gastric cancer: disease or pseudo-disease?. *Lancet*.

[46] Japanese Research Society for Gastric Cancer (1973). The general rules for the gastric cancer. *Japanese Journal of Surgery*.

[47] Akopyanz N, Bukanov NO, Westblom TU, Kresovich S, Berg DE (1992). DNA diversity among clinical isolates of *Helicobacter pylori* detected by PCR-based RAPD fingerprinting. *Nucleic Acids Research*.

[48] Han FC, Ng HC, Ho B (2003). Stability of randomly amplified polymorphic DNA fingerprinting in genotyping clinical isolates of *Helicobacter pylori*. *World Journal of Gastroenterology*.

[49] Röesler BM, De Oliveira TB, Bonon SHA, Monici LT, Zeitune JMR, Costa SCB (2009). Restriction fragment length polymorphism of urease C and urease B genes of *Helicobacter pylori* strains isolated from brazilian patients with peptic ulcer and chronic gastritis. *Digestive Diseases and Sciences*.

[50] Censini S, Lange C, Xiang Z (1996). cag, a pathogenicity island of *Helicobacter pylori*, encodes type I-specific and disease-associated virulence factors. *Proceedings of the National Academy of Sciences of the United States of America*.

[51] Franco AT, Johnston E, Krishna U (2008). Regulation of gastric carcinogenesis by *Helicobacter pylori* virulence factors. *Cancer Research*.

[52] Blaser MJ, Perez-Perez GI, Kleanthous H (1995). Infection with *Helicobacter pylori* strains possessing cagA is associated with an increased risk of developing adenocarcinoma of the stomach. *Cancer Research*.

[53] Wang SK, Zhu HF, He BS (2007). CagA+ H pylori infection is associated with polarization of T helper cell immune responses in gastric carcinogenesis. *World Journal of Gastroenterology*.

[54] Odenbreit S, Püls J, Sedlmaier B, Gerland E, Fischer W, Haas R (2000). Translocation of *Helicobacter pylori* CagA into gastric epithelial cells by type IV secretion. *Science*.

[55] Higashi H, Tsutsumi R, Muto S (2002). SHP-2 tyrosine phosphatase as an intracellular target of *Helicobacter pylori* CagA protein. *Science*.

[56] Franco AT, Israel DA, Washington MK (2005). Activation of *β*-catenin by carcinogenic *Helicobacter pylori*. *Proceedings of the National Academy of Sciences of the United States of America*.

[57] Murata-Kamiya N, Kurashima Y, Teishikata Y (2007). *Helicobacter pylori* CagA interacts with E-cadherin and deregulates the *β*-catenin signal that promotes intestinal transdifferentiation in gastric epithelial cells. *Oncogene*.

[58] Amieva MR, Vogetmann R, Covacci A, Tompkins LS, Nelson WJ, Falkow S (2003). Disruption of the epithelial apical-junctional complex by *Helicobacter pylori* CagA. *Science*.

[59] Suzuki M, Mimuro H, Suzuki T, Park M, Yamamoto T, Sasakawa C (2005). Interaction of CagA with Crk plays an important role in *Helicobacter pylori*-induced loss of gastric epithelial cell adhesion. *Journal of Experimental Medicine*.

[60] Bagnoli F, Buti L, Tompkins L, Covacci A, Amieva MR (2005). *Helicobacter pylori* CagA induces a transition from polarized to invasive phenotypes in MDCK cells. *Proceedings of the National Academy of Sciences of the United States of America*.

[61] Pacheco AR, Proença-Módena JL, Sales AIL (2008). Involvement of the *Helicobacter pylori* plasticity region and cag pathogenicity island genes in the development of gastroduodenal diseases. *European Journal of Clinical Microbiology and Infectious Diseases*.

[62] Roesler BM, Costa SCB, Zeitune  JMR, Tonino P (2011). Virulence factors of *Helicobacter pylori* and their relationship with the development of early and advanced distal intestinal type gastric adenocarcinoma. *Gastritis and Gastric Cancer—New Insights in Gastroprotection, Diagnosis and Treatments*.

[63] Atherton JC (2006). The pathogenesis of *Helicobacter pylori*-induced gastro-duodenal diseases. *Annual Review of Pathology*.

[64] Cover TL, Blanke SR (2005). *Helicobacter pylori* VacA, a paradigm for toxin multifunctionality. *Nature Reviews Microbiology*.

[65] Kusters JG, Van Vliet AHM, Kuipers EJ (2006). Pathogenesis of *Helicobacter pylori* infection. *Clinical Microbiology Reviews*.

[66] Gebert B, Fischer W, Weiss E, Hoffmann R, Haas R (2003). *Helicobacter pylori* vacuolating cytotoxin inhibits T lymphocyte activation. *Science*.

[67] Atherton JC, Cao P, Peek RM, Tummuru MKR, Blaser MJ, Cover TL (1995). Mosaicism in vacuolating cytotoxin alleles of *Helicobacter pylori*. Association of specific vacA types with cytotoxin production and peptic ulceration. *Journal of Biological Chemistry*.

[68] Letley DP, Lastovica A, Louw JA, Hawkey CJ, Atherton JC (1999). Allelic diversity of the Helicobacter priori vacuolating cytotoxin gene in South Africa: rarity of the vacA s1a genotype and natural occurrence of an s2/m1 allele. *Journal of Clinical Microbiology*.

[69] Rhead JL, Letley DP, Mohammadi M (2007). A new *Helicobacter pylori* vacuolating cytotoxin determinant, the intermediate region, is Associated with gastric cancer. *Gastroenterology*.

[70] Sugimoto M, Yamaoka Y (2009). The association of vacA genotype and *Helicobacter pylori*-related disease in Latin American and African populations. *Clinical Microbiology and Infection*.

[71] Kudo T, Nurgalieva ZZ, Conner ME (2004). Correlation between *Helicobacter pylori* OipA Protein Expression and oipA Gene Switch Status. *Journal of Clinical Microbiology*.

[72] Dossumbekova A, Prinz C, Mages J (2006). *Helicobacter pylori* HopH (OipA) and bacterial pathogenicity: genetic and functional genomic analysis of hopH gene polymorphisms. *Journal of Infectious Diseases*.

[73] Dossumbekova A, Prinz C, Gerhard M (2006). *Helicobacter pylori* outer membrane proteins and gastric inflammation. *Gut*.

[74] Yamaoka Y, Kwon DH, Graham DY (2000). A Mr 34,000 proinflammatory outer membrane protein (oipA) of *Helicobacter pylori*. *Proceedings of the National Academy of Sciences of the United States of America*.

[75] Nardone G, Morgner A (2003). *Helicobacter pylori* and gastric malignancies. *Helicobacter*.

[76] Machado JC, Figueiredo C, Canedo P (2003). A proinflammatory genetic profile increases the risk for chronic atrophic gastritis and gastric carcinoma. *Gastroenterology*.

[77] Hou L, El-Omar EM, Chen J (2007). Polymorphisms in Th1-type cell-mediated response genes and risk of gastric cancer. *Carcinogenesis*.

[78] Wex T, Ebert MPA, Kropf S (2008). Gene polymorphisms of the NOD-2/CARD-15 gene and the risk of gastric cancer in Germany. *Anticancer Research*.

[79] Santini D, Angeletti S, Ruzzo A (2008). Toll-like receptor 4 Asp299Gly and Thr399Ile polymorphisms in gastric cancer of intestinal and diffuse histotypes. *Clinical and Experimental Immunology*.

[80] Pandey R, Misra V, Misra SP, Dwivedi M, Kumar A, Tiwari BK (2010). *Helicobacter pylori* and gastric cancer. *Asian Pacific Journal of Cancer Prevention*.

[81] Ngoan LT, Mizoue T, Fujino Y, Tokui N, Yoshimura T (2002). Dietary factors and stomach cancer mortality. *British Journal of Cancer*.

[82] Chao A, Thun MJ, Jane Henley S, Jacobs EJ, McCullough ML, Calle EE (2002). Cigarette smoking, use of other tobacco products and stomach cancer mortality in US adults: the cancer prevention study II. *International Journal of Cancer*.

[83] Nishino Y, Inoue M, Tsuji I (2006). Tobacco smoking and gastric cancer risk: an evaluation based on a systematic review of epidemiologic evidence among the Japanese population. *Japanese Journal of Clinical Oncology*.

[84] Ladeiras-Lopes R, Pereira AK, Nogueira A (2008). Smoking and gastric cancer: systematic review and meta-analysis of cohort studies. *Cancer Causes and Control*.

[85] Steevens J, Schouten LJ, Goldbohm RA, Van Den Brandt PA (2010). Alcohol consumption, cigarette smoking and risk of subtypes of oesophageal and gastric cancer: a prospective cohort study. *Gut*.

[86] Sung JJY, Lin SR, Ching JYL (2000). Atrophy and intestinal metaplasia one year after-cure of *H. pylori* Infection: a prospective, randomized study. *Gastroenterology*.

[87] Leung WK, Sung JJY (2002). Review article: intestinal metaplasia and gastric carcinogenesis. *Alimentary Pharmacology and Therapeutics*.

[88] Correa P, Fontham ETH, Bravo JC (2000). Chemoprevention of gastric dysplasia: randomized trial of antioxidant supplements and anti-*Helicobacter pylori* therapy. *Journal of the National Cancer Institute*.

[89] Mera R, Fontham ETH, Bravo LE (2005). Long term follow up of patients treated for *Helicobacter pylori* infection. *Gut*.

[90] Take S, Mizuno M, Ishiki K (2005). The effect of eradicating *Helicobacter pylori* on the development of gastric cancer in patients with peptic ulcer disease. *American Journal of Gastroenterology*.

[91] Kamada T, Hata J, Sugiu K (2005). Clinical features of gastric cancer discovered after successful eradication of *Helicobacter pylori*: results from a 9-year prospective follow-up study in Japan. *Alimentary Pharmacology and Therapeutics*.

[92] Takenaka R, Okada H, Kato J (2007). *Helicobacter pylori* eradication reduced the incidence of gastric cancer, especially of the intestinal type. *Alimentary Pharmacology and Therapeutics*.

[93] Ogura K, Hirata Y, Yanai A (2008). The effect of *Helicobacter pylori* eradication on reducing the incidence of gastric cancer. *Journal of Clinical Gastroenterology*.

[94] Wong BCY, Lam SK, Wong WM (2004). *Helicobacter pylori* eradication to prevent gastric cancer in a high-risk region of
china: a randomized controlled trial. *Journal of the American Medical Association*.

[95] Mabe K, Takahashi M, Oizumi H (2009). Does *Helicobacter pylori* eradication therapy for peptic ulcer prevent gastric cancer?. *World Journal of Gastroenterology*.

[96] Fuccio L, Zagari RM, Minardi ME, Bazzoli F (2007). Systematic review: *Helicobacter pylori* eradication for the prevention of gastric cancer. *Alimentary Pharmacology and Therapeutics*.

[97] Cheung TK, Wong BCY (2008). Treatment of *Helicobacter pylori* and prevention of gastric cancer. *Journal of Digestive Diseases*.

[98] Herrera V, Parsonnet J (2009). *Helicobacter pylori* and gastric adenocarcinoma. *Clinical Microbiology and Infection*.

[99] Ono H, Kondo H, Gotoda T (2001). Endoscopic mucosal resection for treatment of early gastric cancer. *Gut*.

[100] Fukase K, Kato M, Kikuchi S (2008). Effect of eradication of *Helicobacter pylori* on incidence of metachronous gastric carcinoma after endoscopic resection of early gastric cancer: an open-label randomized controlled trial. *Lancet*.

[101] Maehata Y, Nakamura S, Fujisawa K (2012). Long-term effect of *Helicobacter pylori* eradication on the development of metachronous gastric cancer after endoscopic resection of early gastric cancer. *Gastrointestinal Endoscopy*.

[102] Matsuo T, Ito M, Tatsugami M (2012). Gastric cancer development after *Helicobacter pylori* eradication therapy: a new form of gastric neoplasia. *Digestion*.

[103] Oda T, Murakami K, Nishizono A, Kodama M, Nasu M, Fujioka T (2002). Long-term *Helicobacter pylori* infection in Japanese monkeys induces atrophic gastritis and accumulation of mutations in the p53 tumor suppressor gene. *Helicobacter*.

